# A Systematic Treatise, Historical, Etiological and Practical, on the Principal Diseases of the Interior Valley of North America, as They Appear in the Caucasian, African, Indian, and Esquimaux Varieties of Its Population

**Published:** 1850-06

**Authors:** 


*A Systematic Treatise, Historical, Etiological and Practical, on the Principal Diseases of the Interior Valley of North America, as they appear in the Caucasian, African, Indian, and Esquimaux Varieties of its Population.* By Daniel Drake, M. D. Cincinnati, Winthrop B. Smith & Co. Philadelphia, Grigg, Elliott & Co. New York, Mason & Law. 1850. pp. 878. 8vo. *With Hydrographical Map and Diagrams, and numerous Topographical Maps and Plans of Cities.*

How different from what it now is, and how greatly improved would be medical literature and the practice of medicine at the present day, if the precepts of Hippocrates had been carried out by his successors, after the example which that eminent philosopher and teacher himself gave, in his treatise " *On Airs, Waters, and*

*Places*."* Whoever, he says, wishes to investigate medicine properly, should in the first place consider the seasons of the year, and what effects each of them produces; then the prevalence and temperature of the winds, distinguishing those of perennial direction from those which are peculiar to each locality, and the qualities of the waters. In the same manner, when one comes into a city or district in which he is a stranger, he ought to consider its situation, its exposure to the winds and to the sun, the waters which the inhabitants use, the nature of the ground, whether naked and dry, or wooded and well watered, the situation, hollow and confined, or elevated and cold. Next the new comer is to inquire into the mode of living of the inhabitants, and their pursuits, as to whether they are fond of drinking and eating to excess, and given to indolence, or are fond of exercise and labor and not given to excess in eating and drinking.

Informed of these things, a physician "cannot miss knowing, when he comes into a strange city, either the diseases peculiar to the place, or the particular nature of common diseases, so that he will not be in doubt as to the treatment of the diseases, or commit mistakes, as is likely to be the case, provided one had not previously considered these matters. And in particular, as the season advances, he can tell what epidemic diseases will attack the city, either in summer or in winter, and what each individual will be in danger of experiencing from the change of regimen."

A physician, who has thus investigated the circumstances in which he is placed, "must succeed in the preservation of health, and be by no means unsuccessful in the practice of his art."

Just in proportion as the recommendations and example of Hippocrates, in this celebrated treatise, have been followed, has been the permanent reputation of medical writers. To the worthy followers of the Coan sage, we are proud to add the name of Drake, whose labors in the same field of observation will insure him enduring fame, as the faithful and observant historian of the climatic peculiarities, and the medical topography and diseases of so important a portion of the earth as the interior valley of North America, and of the regimen and pathological tendencies and diseases

* The Genuine Works of Hippocrates—Translated from the Greek—with a Preliminary Discourse and Annotations. By Francis Adams, LL. D. In two volumes. London. Published by the Sydenham Society.

of the different races by which it is inhabited. The physiological and hygienic comparisons instituted by Hippocrates between the people of Asia and Europe,—families of the same race,—are made on a scale of greater geographical extent, and on various races by the American writer, with greater precision and accuracy of detail, obtained by the aid of scientific investigations and appliances which were unknown to the great master of Greek medicine.

We cannot, in advance of any critical or even analytic view of Dr. Drake's treatise, give a better idea of our appreciation of its great and distinctive merits than in thus connecting it with the treatise of Hippocrates "on Airs, Waters, and Places."

Having once entered on the field of investigation which he had proposed to himself, Dr. Drake continued to prosecute his inquiries and observations for a term of years, with perseverance, and a sincere desire to arrive at a knowledge of the facts and without any preconceived theory or bias.

The volume before us consists of two Books, the first of which contains three parts; of these latter, the first has sixteen chapters, the second five, and the third four chapters. Book second includes eleven chapters.

Book first treats of *General Etiology*, which is discussed under the heads or parts of 1. *Topographical and Hydrographical Etiology; and* 2. *Climatic Etiology.* The second, which is the most attractive theme, cannot, however, be either studied or understood to any extent without the aid of the first; and the two jointly are of paramount value, we might say necessity, for a clear knowledge of the diseases described in a subsequent part of the work. It would be a grave mistake in the reader and injustice to the author, not to see a unity of plan and purpose in its numerous divisions. Many of these, which, at first sight, would seem to be matters of allowable philosophical curiosity merely, or at most collateral or even introductory, are, truly, an integral and indispensible part of the treatise; and they could not be detached from it without marring its proportions, if not actually destroying its characteristic and attractive features.

The common constituents of climate, in temperature, atmospheric pressure, and dryness and moisture, force and direction of the winds, and electricity, are greatly modified, independently of latitude and the succession of seasons, by the localities, whose peculiarities are included under the head of *Topographical and Hydrographical Etiology.* This is first exhibited in a "*general analysis*," and then in connection with successive portions of the "*southern hydrographical basin*," which includes the Gulf of Mexico and the different towns and harbours and inlets from Vera Cruz to Mobile, and the *Delta of the Mississippi.* Thence, in one direction, we follow along the bottoms and bluffs of this river on to the Upper Mississippi, and, in another line, the regions "*West of the Gulf and of the Mississippi River.*" In fine, the entire regions on each side of the father of waters, up to the Ohio Basin and the countries north of this latter, are spread before us by the author, who has thus collected and concentrated an amount of medical hydrography and topography of the great valley of the Mississippi, hitherto either unknown, or to most of us unattainable.

Next we are taken over the "*Eastern* or *St. Lawrence Basin*," including the regions of the lakes and of the river itself; and the first part is concluded by a sketch of the "*Hudson and Arctic Hydrographical Basins.*"

Part second is devoted to "*Climatic Etiology*" under its appropriate divisions. Part third furnishes us with "*Physiological and Social Etiology*," especially of the Caucasian portion of the people of the Great Valley, in their physiological characteristics, diet and use of stimulating and narcotic substances; also in their clothing, lodging, bathing and habitations, and in their occupations, pursuits, exercise and recreations. The Hippocratic advice given at the beginning of this notice, has been fully followed out by Dr. Drake; and no physician can henceforward pretend to practice medicine in the great valley of North America, without having first possessed himself of the Systematic Treatise and studied carefully its contents, that so he may prepare himself for a suitable treatment of the diseases in his town or district, and for filling up from his own observation the great outlines furnished in this work.

Book second exhibits the application of all that preceded it to a review of the geographical and topographical, in fine, the climatic causes of Febrile Diseases; beginning with Autumnal Fever, being that which has the widest range, and attacks the greatest number of people. The divisions of this latter described by Dr. Drake, are first, Intermittent Fever, with its varieties of simple, inflammatory and malignant, and then Remittent Fever, considered as simple and malignant. The last two chapters are given to a consideration of the "*Pathological Anatomy and Consequences of Autumnal Fever.*"

From the preceding outline our readers may acquire some idea of the abundance and variety of the topics examined in the present volume. Of their richness and intrinsic worth, they can only obtain accurate notions by reference to its pages. Even if more space were allotted to us than is possible within the assigned limits of this journal, we should still fail to do justice to the multiplied and pains-taking researches, statistical and other tabular estimates, as of temperature, barometrical changes and seasons, interspersed with judicious summaries and apposite statements of the material facts, with which this volume abounds.

Adequate apology, if any be needed, for the want, on the part of the author, of more abundant literary reference and comparisons between the American and other climates, is given in the following passage of his Preface, in which he says:

"Long journeys of observation, repeated through a large part of several years, with elementary teaching in winter, have much abridged the time for bibliothecal research, and, perhaps, even diminished the taste for that mode of inquiry." After admitting that, notwithstanding his extensive explorations, large regions of country remain unvisited, he adds that, "as his personal examinations were carried through eighteen degrees of latitude and as many of longitude, he trusts that facts which may, in some degree, stand as representatives of the whole, *have* been collected; and, therefore, that no general conclusion will be found radically wrong."

While prosecuting his researches, the author "visited the larger part of the military and naval posts of the Interior Valley, both American and British, bearing a letter explanatory of his object from Major General Scott, and received, from each, such facilities as were practicable." We can readily understand the value of these visits, from the fact that at the American posts, for many years past, meteorological registers have been carefully kept, agreeably to rules and instructions laid down for the purpose when Mr. Calhoun was Secretary of War.

The engravings, for the illustration of medical topography, include those of the Bay of Pensacola, Mobile Bay and City, the Delta of the Mississippi, New Orleans, Transverse Section of the Trough of the Mississippi, Memphis, with a Section of the Trough of the Mississippi, St. Louis, Louisville, Harroldsburg Springs, Pittsburg, Cincinnati, Mackinac, Buffalo, Island of Montreal and Quebec. Not only are there engravings of the cities just named, but, also, of the environs of each. Accompanying them are brief but lucid descriptions of the soil and of the geological formation of the adjacent region, either maritime or alluvial, together with their peculiar climatic features.

Not the least attractive subject, of those which come under a consideration of the influences of climate, is that of "Climatic Distribution of Plants and Animals," treated of in Chapter V. of Part Second.

We should like much to be able to present a summary, with illustrative extracts, of the Physiological and Social Etiology, contained in Part third of Dr. Drake's volume; but our limits forbid. The reader will find a pleasant notice of the different national origins of the people of the great valley and of the late and present emigrants from Europe, coming from no less than seven different countries; the first enumerated being the Irish, and the last the Poles. The oldest great family of all, that of the Jews, is also mentioned.

In the chapter on Physiological Characteristics, under the head of Intermarriage, our attention was arrested by the following paragraphs:

"1. Our frontiers, from Quebec, round by the Lakes and Hudson Bay, to the Gulf of Mexico, beyond the Rio del Norte, present a mixed race of whites and Indians; which is gradually lost, in the population residing immediately *within* that boundary. Thus Indian blood is, as it were, absorbed by the surface of the new nation. The readiest amalgamation with the people of that race, is by the northern French, and the southern Spanish Creoles; but the Anglo-American immigrants from the Atlantic States, and their descendants have, at all times, when war did not prevent it, shown a propensity of the same kind.

"2. Wherever there is a negro population, bond or free, the same coalescence is displayed; so that in all our towns, from Mobile to Montreal, and from Pittsburg to St. Louis, the streets are more or less thronged with mulattoes, quadroons, and other mixed breeds, all pressing upward, that is, ambitious of intermarriage with those whiter than themselves; and thus our Caucasian blood is constantly, though slowly, acquiring an African element. In the willingness for this commingling, the Spanish Creoles of Florida, Louisiana and Mexico, stand first; next come the French Creoles of the Lower Mississippi; then some of the classes of the modern emigrants from Great Britain, Ireland and Germany; lastly, the native Anglo-Americans."

Apposite remarks, respecting the changes being wrought on the inhabitants of the great valley, of which intermarriage is one, are made under the additional heads of changes of climate, of food, and of political, moral and social condition.

The section on statistical physiology communicates some precise returns respecting the stature, weight and strength of the different people who inhabit the valley. Towards the illustration of this subject the materials are as yet few.

Instructive matter is given in the chapter on the modes of living of the people of the great valley, including a consideration of the solid and liquid food and table drinks. The varieties of water are considered, both natural for drink, mineral for medicinal uses, and artificial for both purposes.

When speaking of alcoholic drinks, the author tells us that "the successful cultivation of the vine in different parts of the valley, above all in the country around Cincinnati, has originated the manufacture of wine—not from various ingredients—but from the unadulterated juice of the grape."

"Bar Room Drinking.—While family and hospitable drinking have thus declined, bar room drinking, in many parts of the valley, has held its own."

Large quantities of beer "are drunk in the beer houses with which all our towns and cities abound. It is the cherished beverage of our German and English immigrant population. Cider, formerly manufactured and consumed in large quantities by the people of the State of Ohio, is now in less general use.

Dr. Drake discusses the question of the "Necessity and Effects of Alcoholic Drinks." He shows from *a priori* reasoning and from the evidence of facts on a large scale, that there exists no such necessity as that claimed for their use by some physiological writers. He admits that there is a desire for stimulants as well as a desire for food implanted in the physical system of man. These are common salt, an element of the blood, not less than a stimulus; the various aromatic and acrid substances, which man has sought out and instinctively mingled with his food and drinks, under the name of condiments; and, lastly, tea and coffee. These are all the physical stimulants which his system demands for its full perfection—all that are necessary to satisfy his desires, when kept unperverted. Adapted by infinite wisdom, to man's wants, not less than to his instincts and appetites, he seldom uses them to excess; and when he does, their injurious effects bear no assignable proportion to those of the artificial substitutes, which his ingenuity has manufactured out of sugar, now known to be the only source of alcohol." In these conclusions we heartily concur.

Tobacco is largely used in the great valley, as, unfortunately, it is everywhere else. The author thinks that "nothing in the present state of society justifies the expectation that tobacco will go out of use."

When speaking of "Clothing," the author tells us that the male population of the Valley are generally well clothed, as far as relates to protection from cold. The same cannot be said of the dress of the females, in which cotton and silk too often exclude woollen textures. An improvement, however, is going on in this respect, especially in the more general use of muslin or flannel drawers. Proper strictures are made on the dress of the children, in their being often inadequately clothed—not from deficiency of means on the part of their parents, but to meet the requirements of absurd fashions. Specification is made of the pernicious fashion of exposing the arms and upper parts of the chest during youth.

Bathing, we regret to learn, is far from being general in any part of the Valley. "Our large cities, from New Orleans and Mobile to Pittsburg and Montreal, ought to have public cistern-baths, for the gratuitous accommodation of the poorer laboring classes, so many of whom, when sick, are supported at the public expense in our almshouses and hospitals." It would be easy, as Dr. Bell has shown in his late work on Baths and the Watery Regimen, &c., to procure warm baths on a large scale, at a low cost, wherever steam power is used.

Judicious remarks follow on Lodgings, Habitations and Shade Trees; and, in successive chapters, on Agricultural Labor, Commercial Pursuits, Mining and Smelting, Salt Making, Mechanical and Chemical Arts and Manufactures, in reference to the health and tendency to disease of those engaged in them.

Under the head of Exercises, Recreation and Amusement, Dr. Drake lays merited stress on the necessity of a portion of time being allotted to these purposes, in the interests of a healthful state of both body and mind. He regrets that the utility of systematic exercise is so little appreciated in the Great Valley, and he shows its importance on physiological grounds. On the subject of Dancing, the author asserts, too truly, that, "As a mode of exercise in childhood and youth, it is insufficient; and as a method of amusement in after years, it is neglected by those who, physiologically speaking, most require it."

At the conclusion of Book first, the author indulges in some remarks on the probable future, as regards the physiological traits, and the diseases of the people who shall then inhabit the Great Valley.

"The physicians of a future day will see, what we cannot now, a prevailing temperament, a stature, form, complexion and physiognomy, characteristic of an indigenous, but greatly compounded race; with its own physical, intellectual and moral constitution; its special liabilities and exemptions from disease; its national idiosyncrasies, and the required peculiarities of hygienic regimen and therapeutic treatment. In the course of this development, what hereditary diatheses may disappear, and what new ones take their places; what new maladies may arise, or old ones cease or become greatly modified, under the joint influence of mingled blood, of climate, water, occupations, modes of living, customs, and moral, social and political influences, cannot be specified; but a few predictions may be hazarded:

"1. Autumnal fever will decrease, and typhus and typhoid fevers become more prevalent.

"2. Gout will occur oftener than at present.

"3. The diseases produced by the intemperate use of ardent spirits will diminish.

"4. Consumption and scrofula will increase.

"5. Apoplexy, palsy and epilepsy will become more frequent.

"6. Diseases of the liver will become less, and those of the mucous membrane of the bowels more prevalent.

"7. Lastly, mental alienation will be more frequent."

Book Second will be devoted, as we learn at p. 702, "to febrile diseases, under the five following heads: *First*, AUTUMNAL FEVER—*second*, YELLOW FEVER—*third*, TYPHOUS FEVERS—*fourth*, ERUPTIVE FEVERS—*fifth*, PHLOGISTIC FEVERS, or the PHLEGMASIÆ."

The first part of the second Book on Autumnal Fever, with its varieties already designated, is contained in the present ample volume. The article on Yellow Fever, will, as the author informs us in his Preface, make the first part of the second volume, "the materials for which have been chiefly collected, and considerable portions of it written, so that the author hopes it may be committed to press in about a year"—from December, 1849.

We cannot pretend to review the part on Autumnal Fever; but we are safe in recommending it for the ample views which it presents of the etiology and special pathology of the varieties and complications of periodical fever,—although on some points we might express our dissent from the opinions of the author, as, for instance, on the subject of Malaria.

We close reluctantly our notice of this valuable work, of which not only the people of the Great Valley, but also of the United States, may be justly proud, as an enduring contribution, of the highest order, to our national medical literature. Its estimable author may send it to Europe as a rich equivalent for medical productions obtained from that quarter and largely current among us.

A word to our readers. The volume now issued, although but a part of the great work, is so full and complete in its divisions, and these have such connection and co-ordination one with another, that it may be read and studied as an entire production, susceptible of connection with that which is to come, but not dependent on it for harmonious proportions and general fitness and adaptation.

